# Editorial Note: Small object detection algorithm incorporating swin transformer for tea buds

**DOI:** 10.1371/journal.pone.0346115

**Published:** 2026-03-31

**Authors:** The PLOS One Editors

The *PLOS One* Editors issue this Editorial Note for this article [1] because of concerns identified about the peer review process. Readers are advised to interpret the article [1] with caution.

The Editors have no evidence of any author involvement in the peer review concerns.

## References

[1] Shi M, Zheng D, Wu T, Zhang W, Fu R, Huang K. Small object detection algorithm incorporating swin transformer for tea buds. PLoS One. 2024;19(3):e0299902. doi: 10.1371/journal.pone.0299902 38512917 PMC10956868

